# The First Sporadic Creutzfeldt–Jakob Disease Case with a Rare Molecular Subtype VV1 and 1-Octapeptide Repeat Deletion in *PRNP*

**DOI:** 10.3390/v13102061

**Published:** 2021-10-14

**Authors:** Aušrinė Areškevičiūtė, Eva Løbner Lund, Sabina Capellari, Piero Parchi, Christian Tersbøl Pinkowsky

**Affiliations:** 1 Department of Pathology, Danish Reference Center for Prion Diseases, Copenhagen University Hospital, 2100 Copenhagen, Denmark; ausrine.areskeviciute@regionh.dk (A.A.); eva.loebner.lund@regionh.dk (E.L.L.); 2 IRCCS Istituto delle Scienze Neurologiche di Bologna, Ospedale Bellaria, 40123 Bologna, Italy; sabina.capellari@unibo.it (S.C.); piero.parchi@unibo.it (P.P.); 3 Department of Experimental Diagnostic and Specialty Medicine (DIMES), University of Bologna, 40126 Bologna, Italy; 4 Department of Spinal Cord Injuries, Copenhagen University Hospital, 3100 Hornbæk, Denmark

**Keywords:** Creutzfeldt–Jakob disease, 1-OPRD, VV1, sporadic prion disease, prions, 58-year-old female patient, prion protein gene, deletion polymorphism

## Abstract

In the present manuscript, we report the clinical presentation and challenging diagnostic work-up of a sporadic Creutzfeldt–Jakob disease patient with confirmed VV1 subtype and heterozygous 1-octapeptide repeat deletion in the prion protein gene. The described patient was a 58-year-old woman. Interestingly, most of the reported patients with the VV1 subtype to date are men with an average age of 44 years at disease onset. The patient was observed clinically from symptoms onset until her death 22 months later. This report describes the patient’s insidious clinical evolution and the paraclinical examinations and pathology reports gathered at different time points of disease progression. Unfortunately, the absence of typical clinical and paraclinical features of classic sporadic Creutzfeldt–Jakob disease made the brain biopsy surgery necessary. This case report illustrates the diagnostic difficulties posed by the phenotypic heterogeneity of sporadic Creutzfeldt–Jakob disease and urges clinicians to consider this diagnosis even in patients who do not fulfil the typical clinical disease criteria. Furthermore, it highlights the need for real-time quaking-induced conversion method adaptation for detection of rare sporadic Creutzfeldt–Jakob disease subtypes with certain prion protein gene variants.

## 1. Introduction

Sporadic Creutzfeldt–Jakob disease (sCJD) is the most common form of Prion disease, a group of neurodegenerative disorders characterized by self-replicating and aggregating misfolded cellular prion proteins called prions (denoted PrP^Sc^). Although sCJD is much more prevalent (~85%) than the genetic (~15%) and iatrogenic (~1%) disease forms, what triggers the spontaneous misfolding of cellular prion proteins (PrP^C^) in otherwise healthy older individuals remains poorly understood.

Despite the missing depth in our understanding of sCJD etiology, over the last 4 decades scientists have established an elaborate classification system based on the combination of neuropathological and molecular findings in the brains of sCJD patients. Currently, there are 14 different molecular sCJD subtypes that are reviewed elsewhere [1]. However, they all stem from the main six subtypes linked to five different PrP^Sc^ strains [2,3].

The six sCJD subtypes are defined by the patient’s genotype at polymorphic methionine/valine codon 129 in the prion protein gene (*PRNP*) and the type of PrP^Sc^ found in the brain. The type of PrP^Sc^ is determined by the size of its protease K-treated unglycosylated fragment, which, in the case of the main six sCJD subtypes, can either be 21 kDa (type 1 PrP^Sc^) or 19 kDa (type 2 PrP^Sc^). Therefore, the six sCJD subtypes comprise MM1, MV1, VV2, MV2, MM2, and VV1, where MM1/MV1 is the most common (approximately 65% of all sCJD cases), and VV1 is one of the rarest (only 1% of all sCJD cases).

Genetic prion diseases show an even more heterogeneous clinical phenotype, and can be caused by three main types of mutations: Nonsense, missense and octapeptide repeat deletions (OPRD), and insertions (OPRI) in the *PRNP*. Currently, the list of different *PRNP* variants includes approximately 60 mutations. However, the penetrance of different *PRNP* mutations is quite variable, and with enabled large population studies some of the variants were even shown to be not or lowly pathogenic [4,5].

The size and location of the mutation in the *PRNP* and codon 129 genotype seem to play a crucial role in disease pathogenicity, clinical presentation, and neuropathology. Interestingly, in the case of OPRIs, which can include two to 12 extra inserts in the *PRNP* octapeptide repeat region, the reported clinicopathological variability is too wide even within the same family members to confidently characterize OPRIs based only on their length. In contrast, 1-OPRI and 1-OPRD, although present in some CJD patients, do not qualify as pathogenic [6,7,8].

In this report, we describe the clinical manifestation and extensive diagnostic work-up of the first Danish sCJD VV1 case in a patient carrying 1-OPRD in *PRNP*. Furthermore, we discuss how this case compares to other published VV1 cohorts and what could help neurologists reach the final diagnosis faster. The level of clinical details provided in this report of disease manifestation will undoubtedly enrich the currently available medical literature on this topic. In addition, it will expand the spectrum of known clinical symptoms linked to this rare sCJD subtype.

## 2. Case Report

### 2.1. Disease Presentation

At the age of 58, a female patient presented with an insidious debut of symptoms in April 2019, where she described to her mother that she could not read newspapers anymore. She explained that she could read the words, but that the language did not make any sense to her. In August, she could not make herself understandable at work where she was obliged to make short written reports from shifts, at an institution for people with mental disability. She and her social network thought that it was a sign of stress at work. In September, she reported reduced understanding of spoken language and difficulty expressing herself. She was admitted to a hospital on the suspicion of stroke with aphasia. At no point had she experienced disturbance of sight, motor control, paresis, seizures or fainting. She experienced no constitutional symptoms. On examination, she did not show any neurological deficits. She was discharged after a brief treatment with a high-dosage of steroids, which yielded no effect.

The patient was admitted again in October and reported that she could not understand conversations with friends and family and what is being said on television. Furthermore, she reported a failing short-term memory. She reported that life was not worth living and manifested suicidal thoughts, “if nothing could be done”. She denied visual disturbances, sensory disturbances, motor control problems or involuntary movements. Her relatives confirmed all of the above. The family reported that the patient had a regular daily conversation, and continued normal behavior, cooking ability, and sustained fitness training. Upon examination, a snout-reflex was found and the patient continuedly repeated not being able to understand the examiner. There was a reduced vocabulary. Moreover, she was emotionally distraught and prone to crying. She was able to comply with three-stage commands, and no motor-, sensory- or ataxic signs were found. She remained admitted for the next month, in which her cognitive dysfunction suffered significant progression. The patient did not report sight disturbances, including color disturbance, visual field loss, object distortion, blurring, loss of sight or hallucinations. No signs of ataxia, myoclonus, startle response, rigidity or stupor. A neuropsychological evaluation during this admittance revealed reduced verbal and nonverbal learning, narrow working memory, reduced concentration, reduced language comprehension, reduced word mobilization, poor verbal abstraction, and reduced phonological word mobilization.

The patient had a brain biopsy performed at the end of November and, after that, was transferred back to the neurology department, where further cognitive deterioration was evident. She was no longer able to participate relevantly in a neuropsychological evaluation. Her language was perseverant, and she could no longer comply with purely verbal commands. She received sedatives for severe dysphoria. She was discharged to a care facility, which she left on her own and walked directly to her home 15 km away. After that event, she had supervised care in her home.

By February, she could not produce words, but could still care for herself in her home, with a little help from her mother and the municipality.

By May, she started developing myoclonus and her gait was slightly disturbed without any falls. She required help with bathing and hygiene but could still eat and walk on her own. She could no longer utter sentences. The patient could say “yes” and “no” from time to time, but only occasionally seemed to understand what was being said. She appeared regressive, seemed happy, and showed personal items to the examiner, including puzzles, coins, and other collectibles. The patient had a chuckling laugh as the most frequent communication. The chuckling was heard both when she was alone and in the company of her mother and strangers, apparently without distinction.

By October, she had been moved to a care facility. The patient was mobilized via a reclining wheelchair. The patient no longer spoke but had eager attempts at verbalization and frequent laughing. She was fully awake and followed the examiner with her eyes. A brisk, multifocal myoclonus was seen, especially upon motor activation. She had a startle response by then, upon clapping or other sudden sounds. She had help with eating, personal hygiene, mobilization, and dressing.

By January, she was still without any drowsiness. She still sought eye contact. She followed moving objects with the eyes, but predominantly had the head turn to the left. She oriented herself only sporadically towards the sound. Lively, multifocal myoclonus was seen, accentuated by sound, and active as well as passive movement. She had startle response and echopraxia, snout- and grasp reflex. The patient still had some attempts at verbalization and some laughing. During eating, the patient swallowed on reflex but showed no signs of intentional chewing. She developed dysphagia but had been able to maintain her weight until then. A regimen of palliative care was instituted at the time the patient could not participate in eating any longer.

The patient passed away in February, 22 months after the first symptoms showed.

We have listed the symptoms’ progression in Table 1.

### 2.2. Clinical Tests

During the initial diagnostic work-up, a variety of diagnoses were considered and examined. Among them were Hashimoto’s encephalitis, steroid-sensitive encephalitis, toxic encephalitis, celiac disease encephalopathy, neurosyphilis, neuroborreliosis, herpetic encephalitis, paraneoplastic encephalitis, and neurodegenerative diseases, other than Creutzfeldt–Jakob disease.

Cerebrospinal fluid analyses performed repeatedly during the disease course revealed increased leucocyte numbers, neurofilament light polypeptide, and tau protein, the presence of immunoglobulin-oligoclonia, and reduced amyloid-beta protein (Table 2). A real-time quaking-induced conversion analysis (RT-QuIC) gave negative results.

Three cerebral magnetic resonance imaging sessions (MRIs) were performed, including two MRI-neuroaxis. They indicated asymmetric signal changes in the cerebral cortex, scattered in both cerebral hemispheres, predominantly lateral in the temporal lobes and to a lesser extent parietally and frontally, especially on the left side. The involved cortex showed gyral enhancement in FLAIR-sequence and DWI with a corresponding diffusion restriction on the ADC map. The cortex appeared without edema and there was no signal changes in the subcortical white matter in these areas. There was no progression of the MRI-changes during follow-up.

Electroencephalogram (EEG) did not indicate periodic three-phase sharp-wave complexes (PSWC). Taken together, none of the performed clinical tests provided strong evidence for a prion disease, and since the patient clinically deteriorated rapidly, it was decided to take a brain biopsy to verify our suspicion of an atypical CJD. The brain biopsy revealed the presence of PrP^Sc^. The timeline overview of the performed paraclinical tests and their results are provided in Table 2.

### 2.3. Neuropathology and Molecular Disease Subtyping

**Figure 1:**
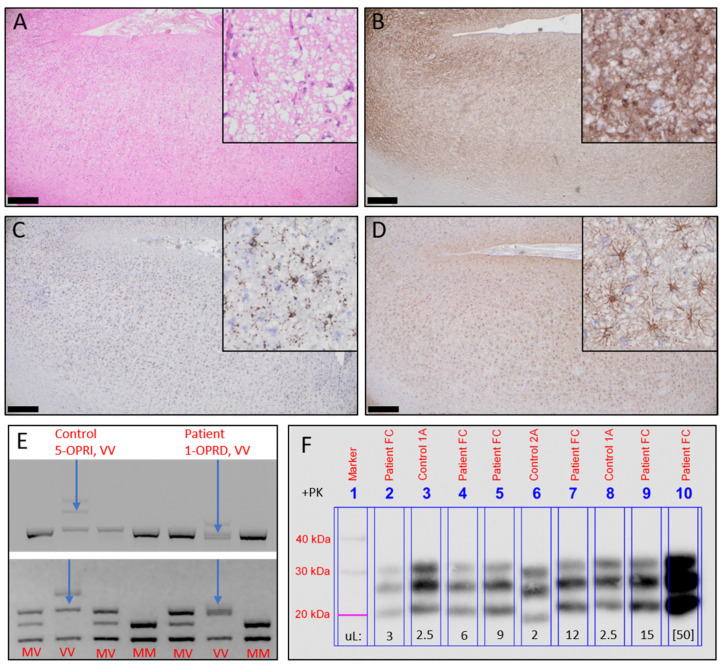
Neuropathology and molecular subtyping. (**A**) Severe cortical spongiosis (H&E staining). (**B**) Diffuse, cortical, protease K-resistant PrP^Sc^ deposits (KG9 immunostaining). (**C**) Cortical microgliosis (CD68 immunostaining). (**D**) Cortical astrogliosis (GFAP immunostaining). Scale bars 500 µm; corner picture frames 200 µm (**E**) (**Top**) Electrophoretic visualization of DG2+I5 PCR products indicating wild type sequences, control sequence with 5-OPRI, and the current case with 1-OPRD. (**Bottom**) Presentation of the DG2+3′SAL PCR product after digestion with XCell demonstrating the electrophoretic patterns of different codon 129 polymorphism variants and indicating that the patient is valine homozygous. (**F**) Western blot analysis of the patient’s brain homogenates showing PrP^Sc^ type 1. Different volumes of the patient’s brain biopsy 10% *w/v* homogenate were treated with proteinase K, run by SDS-PAGE, and immunoblotted with the 3F4 antibody.

A neuropathological examination of the frontal cortex biopsy revealed severe cortical spongiosis, synaptic PrP^Sc^ deposition, pronounced microgliosis, and astrogliosis, which are characteristic features of most molecular subtypes of prion diseases (Figure 1A–D).

The residual biopsy sample was used to determine the molecular disease subtype by *PRNP* coding region amplification, Sanger sequencing, and PCR products’ enzymatic digestion, as well as gel electrophoresis and immunoblotting, as described previously [9,10]. *PRNP* sequencing indicated that the patient had heterozygous 1-octapeptide repeat deletion (1-OPRD, 24bp-del) in the octapeptide repeat region and was valine homozygous at codon 129. The 1-OPRD was confirmed by the presence of two distinct *PRNP* PCR product bands of slightly different sizes. Moreover, the valine homozygosity was confirmed by *PRNP* PCR product digestion with XCell and visualization of its fragments on the agarose gel (Figure 1E: Top and bottom, respectively). The Western blot analysis revealed PrP^Sc^ type 1, which completed the final diagnosis of sCJD subtype VV1 with 1-OPRD (Figure 1F).

## 3. Discussion

This case report provides detailed clinicopathological and biochemical characteristics of sCJD subtype VV1, which is one of the rarest CJD subtypes in the world and is observed in Denmark for the first time.

Moreover, the reported patient carried a heterozygous 1-OPRD in *PRNP*, which is considered a non-pathogenic polymorphism also found in healthy individuals [6,7]. It was even suggested that the presence of 1-OPRD may offer resistance to certain prion strains [11]. On the other hand, with rare sCJD subtypes, similar to the one described here, it is difficult to evaluate the potential impact of 1-OPRD on disease susceptibility, pathogenicity or its phenotype modifying effects.

Furthermore, the Danish patient was a woman, which, with the currently available statistics, seems to be a rare event given that out of nine sCJD VV1 patients in a cohort, eight were men [12]. In addition, the patient’s age at disease onset (58 years) was older than in most of the cases reported to date. The average patient’s age at sCJD VV1 symptoms onset is 44 years, ranging from 19 to 55 years, except for another unusual case in a 79-year-old female [1,13].

Typical differential sCJD clinical signs are rapid disease progression and short duration, which for the most common disease subtypes is approximately 6 months. However, patients with the VV1 subtype show an average disease duration of 21 months, ranging from 17 to 42 months, making an early clinical suspicion of sCJD more challenging. The disease duration of the patient reported here was 22 months, which perfectly fit the statistic [12].

A clinical presentation of patients with sCJD VV1 is characterized by slowly developing dementia and psychiatric disturbances followed by ataxia, rigidity, myoclonus, and spastic tonus increase at the latter stage of the disease [12]. The initial disease symptoms in the Danish patient with sCJD VV1 were also cognitive, but the motor symptoms appeared late in the disease course, and only included myoclonus, startle, and mutism. However, consistent with neuropathological findings, the cerebellum is relatively spared in these cases, and thus ataxia may not be prominent [2].

EEG PSWC, a typical feature in sCJD MM1, the most common sCJD subtype, were lacking in most of the reported sCJD VV1 cases, as well as in our case. A brain MRI in sCJD VV1 most often shows a cortical signal increase, mainly in the frontal and temporal lobes, which is also compatible with the MRI changes observed in the Danish patient [12].

With regards to the CSF biomarker results, the finding of oligoclonal bands in the cerebrospinal fluid, is considered unusual in sCJD, but it does not exclude the diagnosis [14]. The presence of increased CSF levels of protein 14-3-3 was reported in most of the previous VV1 cases. However, its presence was not assessed in the current case [12]. The negative RT-QuIC test is probably explained by its relatively lower sensitivity for the VV1 subtype, which is approximately 75%, as reported by Green in 2018 [15]. Furthermore, it is unknown if the lack of eight amino acids in the PrP^Sc^ (the result of 1-OPRD in *PRNP*) could have also interfered with RT-QuIC sensitivity.

From the available sCJD VV1 case report and cohort studies, it seems that MRI changes in the temporal lobe and a positive 14-3-3 CSF assay represent the most useful biomarkers in support of the clinical diagnosis of this rare sCJD subtype. Nevertheless, the most assuring biomarker in prion disease diagnostics is PrP^Sc^. Therefore, to avoid invasive brain sampling surgeries, it will be important to focus on further improving the RT-QuIC sensitivity for the VV1 subtype.

Diagnosing sCJD can be challenging, especially when encountering its rare subtypes, which do not have the typical CJD clinical development or paraclinical test results [16]. This case report illustrates the tremendous effort currently needed to reach an *antemortem* diagnosis of certain atypical cases of prion disease. Furthermore, it highlights the need for improved, less invasive, early diagnostic approaches capable of detecting even rare disease subtypes with unique polymorphic variants in the *PRNP*.

## Figures and Tables

**Table 1:** Clinical symptoms progression since disease debut.

	Symptom/Finding	2019 April	2019 August	2019 September	2019 October	2019 November	2019 December	2020 February	2020 May	2020 December	2021 January
Cognitive	Depression	-	-	Yes	Yes	Yes	-	-	-	-	-
	Behavioral change	-	-	-	-	-	Yes	Yes	Yes	Yes	Yes
	Reduced intellect	-	-	Yes	Yes	Yes	Yes	Yes	Yes	Yes	Yes
	Apraxia	-	-	-	-	-	-	Yes	Yes	Yes	Yes
	Aphasia	-	-	-	Yes	Yes	Yes	Yes	Yes	Yes	Yes
	Memory loss	-	-	-	Yes	Yes	Yes	Yes	Yes	Yes	Yes
Visual	Color sight change	-	-	-	-	-	-	-	-	-	-
	Visual field loss	-	-	-	-	-	-	-	-	-	Yes
	Object distortion	-	-	-	-	-	-	-	-	-	-
	Blurred vision	-	-	-	-	-	-	-	-	-	-
	Cortical blindness	-	-	-	-	-	-	-	-	-	-
	Hallucinations	-	-	-	-	-	-	-	-	-	-
Motor	Ataxia	-	-	-	-	-	-	-	-	-	-
	Myoclonus	-	-	-	-	-	-	-	Yes	Yes	Yes
	Startle	-	-	-	-	-	-	-	-	Yes	Yes
	Mutism	-	-	-	-	-	Yes	Yes	Yes	Yes	Yes
	Rigidity	-	-	-	-	-	-	-	-	-	-
	Stupor	-	-	-	-	-	-	-	-	-	-

**Table 2:** Overview of the performed paraclinical tests.

CSF Analyses	Ref. Interval	2019 September	2019 October	2019 November	2020 January
Leukocytes	<3 × 10^6^/L	2	2	1	3 (H)
Protein	0.15–0.45 g/L	0.39	0.41	0.41	0.356
Immunglobulin-oligoclonia	-	Present			
Amyloid beta-protein	>1.000 ng/L	1.287	578 (L)	1.016	670 (L)
Phosphorylated tau	<30 ng/L	15	14	15	18
Neurofilament light polypeptide	<890 ng/L		1.837 (H)		8.480 (H)
Tau protein	<300 ng/L	881	1.305 (H)	1.532 (H)	2.310 (H)
RT-QuIC			Negative		
**Changes in MRI**		Yes	Yes	Yes	
**Triphasic EEG**		No	No	No	
**Brain biopsy**				PrP^Sc^ positive	
